# MeCP2 as a genome-wide modulator: the renewal of an old story

**DOI:** 10.3389/fgene.2012.00181

**Published:** 2012-09-11

**Authors:** Floriana Della Ragione, Stefania Filosa, Francesco Scalabrì, Maurizio D’Esposito

**Affiliations:** ^1^Institute of Genetics and Biophysics “A. Buzzati-Traverso”Naples, Italy; ^2^Istituto Di Ricovero e Cura a Carattere Scientifico NeuromedPozzilli, Italy

**Keywords:** MECP2, Rett syndrome, chromatin, DNA methylation, epigenetics

## Abstract

Since the discovery of MeCP2, its functions have attracted the interest of generations of molecular biologists. Its function as a transducer of DNA methylation, the major post-biosynthetic modification found throughout genomes, and its association with the neurodevelopmental disease Rett syndrome highlight its central role as a transcriptional regulator, and, at the same time, poses puzzling questions concerning its roles in physiology and pathology. The classical model of the MeCP2 function predicts its role in gene-specific repression through the binding of methylated DNA, via its interaction with the histone deacetylases and co-repressor complexes. This view has been questioned and, intriguingly, new roles for MeCP2 as a splicing modulator and as a transcriptional activator have been proposed. Recent data have demonstrated that MeCP2 is extremely abundant in the neurons, where it reaches the level of histone H1; it is widely distributed, tracking the methylated CpGs, and regulates repetitive elements expression. The role of MeCP2 in maintaining the global chromatin structure is further sustained by its involvement in other biologically relevant phenomena, such as the Line-1 repetitive sequences retrotransposition and the pericentromeric heterochromatin clustering during cellular differentiation. These new concepts renew the old view suggesting a role for DNA methylation in transcriptional noise reduction, pointing to a key role for MeCP2 in the modulation of the genome architecture.

## MeCP2 AND DNA METHYLATION: IN LIMINE

In 2012, the twentieth anniversary of MeCP2 protein identification will be celebrated (Lewis et al., 1992). The impulse that this discovery gave to research in various, often apparently unrelated biological fields, from gene regulation to medical genetics, has been immense. Here we cannot describe the enormous weight of data produced, in 20 years, by an increasing number of teams. Rather, we wish to review current research on the MeCP2 biology starting from older scientific hypotheses.

MeCP2 was the second methyl-CpG-binding protein to be identified, although it was the first to be cloned. In fact, Boyes and Bird (1991) demonstrated that the methyl-CpG-binding protein MeCP1 can mediate the repression of transcription from densely methylated genes. MeCP1 is able to bind various methylated sequences “*in vitro*,” if at least 12 symmetrically methylated CpGs are available. Like many important findings, MeCP2 was discovered “by accident” by Boyes and Bird (1991), who were attempting to identify the factors that bind unmethylated DNA to protect CpG islands from DNA methylation (Clouaire and Stancheva, 2008). Rat MeCP2 had been successfully isolated through its ability to bind methylated substrates. Then, after its purification, its cDNA had been cloned, thus enabling the knowledge of the nucleotide sequence of the first methyl CpG DNA gene (Lewis et al., 1992).

MeCP2 is able to bind at a genome-wide level, with the need of a single, methylated CpG. This weak discrimination is in agreement with its diffuse nuclear signal in rat cells. In mouse cells, given their peculiar heterochromatin organization, the staining is extremely evident in the pericentromeric heterochromatin, closely resembling the distribution of major satellite DNA (Lewis et al., 1992). Mouse satellite DNA is enriched of methylated CpGs, thus explaining the co-localization of MeCP2 with these genomic regions. MeCP2 was the first methyl-binding protein to be biochemically dissected, revealing the presence of a number of functional domains. The most noticeable domains are the methyl-binding domain (Nan et al., 1993), responsible for binding with the methylated cytosines and the transcriptional repression domain (Nan et al., 1997), which mediates the link with the histone modifications (Jones et al., 1998; Nan et al., 1998; Fuks et al., 2003) and the co-repressors. They play a fundamental role in modulating the functions of MeCP2, the main one being, without doubt, the transduction of DNA methylation. These functions fit with an earlier study reporting that the loss of the X-linked methyl-CpG-binding protein 2 (MeCP2) caused embryonic lethality in chimeric mice (Tate et al., 1996). Taken together, these data highlighted the role(s) of MeCP2 as a genome modulator, whose functions are indispensable for life.

DNA methylation is present, in various degrees, from bacteria to invertebrates and vertebrates. It plays a role in defending bacterial genomes from foreign DNA invasion (Hendrich and Tweedie, 2003). Vertebrate genomes are globally methylated, whereas in invertebrate genomes DNA methylation is patchy. DNA methylation is involved in chromatin remodeling in vertebrates, whereas it is often located inside the genes in invertebrates, such as in *D. melanogaster* (Mandrioli, 2007). Its genome-wide pattern, in vertebrates, prompted Bird to hypothesize an association between a global repressive effect of DNA methylation and the increase in gene number, which is evident when switching from invertebrate to vertebrate genomes (Bird, 1995). In fact, a major change in the distribution of DNA methylation occurred at the invertebrate–vertebrate boundary (Tweedie et al., 1997; Hendrich and Tweedie, 2003). Following Bird’s hypothesis, the global repressive effects of DNA methylation may act as an additional mechanism to suppress transcriptional noise together with the acquisition of a nuclear envelope and the arrangement of the chromatin, which mark the prokaryotes/eukaryotes boundary. This is clearly postulated: “*global improvements in the ability to suppress noise will permit an increase in the maximum gene number, allowing more genes to be tolerated*” (Bird, 1995).

Hendrich and Tweedie (2003) added further substance to this hypothesis suggesting that “*to increase the fidelity of DNA methylation-mediated silencing, and to protect against extensive mutation, there was also a coordinate increase in the number and diversity of methyl CpG binding proteins encoded in the proto-vertebrate genome*”. Hendrich and Bird identified a family of methyl-binding protein genes, characterized, similarly to MECP2, by the presence of the methyl-DNA binding domain (MBD). These proteins, called MBD1, MBD2, MBD3, and MBD4 (Hendrich et al., 1999) were all (except for MBD3) characterized by their ability to bind methylated DNA. Only MBD2 and MBD3 were conserved in invertebrates: the ancestral MBD2/3 gene was encoded by a single gene in invertebrate genomes, in contrast to the two separate genes encoded by vertebrates (Hendrich and Tweedie, 2003).

Thus, if a global DNA methylation has been used, by vertebrate genomes, to reduce unscheduled transcription, thereby increasing the gene number, this would similarly provide an evolutionary pressure to increase the number and diversity of the protein(s) capable of repressing transcription through the binding of methylated DNA.

## MECP2 AND RETT SYNDROME

Rett syndrome (RTT) is a sporadic post-natal progressive neurodevelopmental disorder occurring with a frequency of 1/10000–15000 live females births and is considered the second most common cause of mental retardation in females (Rett, 1966; Hagberg et al., 1983). The large majority of cases (99%) are sporadic. In 1999, Zoghbi and colleagues (Amir et al., 1999) were able to associate loss-of-function heterozygous mutations in the MECP2 gene to classical RTT patients. The discovery of the MECP2 mutations underlying RTT was a surprise because the large amount of data, summarized above, makes the association of MECP2 to a monogenic disease astonishing.

Besides the large number of studies on patients, the modeling of RTT in mice has been instrumental in order to elucidate the molecular basis of the disease. Mouse models have also been pivotal in the study of expression profiling alterations, necessary to identify putative MeCP2 target genes. They have helped in the elucidation of many questions of biomedical importance: is RTT a pure neuronal disease? Is MECP2 dosage important for the establishment of a pathogenic status? Is RTT reversible?

Two Mecp2 null mice obtained with Cre-LoxP technology and carrying an ubiquitous deletion, were viable but affected by severe neurological symptoms characteristic of RTT (Chen et al., 2001; Guy et al., 2001). The comparative analysis of knock out and brain selective deletions of Mecp2 suggested that the function of this gene is relevant for the central nervous system (Chen et al., 2001; Guy et al., 2001). Moreover, the deletion of MeCP2 in selected brain regions or neuronal sub-types revealed the presence of specific subsets of null phenotypes, allowing to ascribe to MeCP2 different neuronal-specific functions (Fyffe et al., 2008; Samaco et al., 2009; Chao et al., 2010).

MeCP2 dosage matters: a mouse over-expressing a transgene containing the human MECP2 locus that shows a near twofold MeCP2 expression, showed severe progressive neurological phenotypes (Collins et al., 2004). The effect of MECP2 over-expression has also been observed in humans, where a double dosage of MECP2 causes a severe developmental delay and mental retardation (Lubs et al., 1999). Such evidence suggests that MeCP2 levels must be fine regulated *in vivo* and even a mild over-expression of this gene can have a dramatic effect.

The concept of RTT as a pure neuronal disease has recently been challenged with results implicating the involvement of the glial cells in the pathogenesis of RTT (Ballas et al., 2009; Maezawa et al., 2009; Zoghbi, 2009). More recently, it has been suggested that the microglia may influence the onset and progression of RTT by releasing elevated doses of glutamate, exerting a toxic effect on neurons in a non-cell autonomous fashion (Maezawa and Jin, 2010). Very interestingly, null phenotypes in mouse models can be reversed by the re-insertion of the Mecp2 gene (Collins et al., 2004; Luikenhuis et al., 2004; Jugloff et al., 2008), while its over-expression by twofold is deleterious (Collins et al., 2004; Luikenhuis et al., 2004). An almost complete reversibility of the null phenotypes was obtained after the onset of the symptoms, by removing a stop cassette in the Mecp2 gene by a Cre-mediated excision induced by tamoxifen administration (Guy et al., 2007). These data suggest that the neurological defects caused by Mecp2 mutations can potentially be reversed.

### MeCP2 AND TRANSCRIPTIONAL CONTROL

The apparent dichotomy of MeCP2 functions (genome-wide vs gene-specific regulator) has been widely debated. Transcriptional profiling studies comparing the total brains of RTT patients or mouse models with controls have revealed only subtle differences in gene expression dampening a role for MeCP2 as a global regulator of transcription (Chadwick and Wade, 2007). A number of reports highlighted BDNF as a bona fide target of MeCP2 in rodent systems (Chen et al., 2003; Martinowich et al., 2003). BDNF is a key signaling molecule involved in brain development and plasticity (Greenberg et al., 2009; Cohen-Cory et al., 2010). The mechanism of its transcriptional regulation is, therefore, quite controversial (Dani et al., 2005; Chang et al., 2006).

**FIGURE 1:**
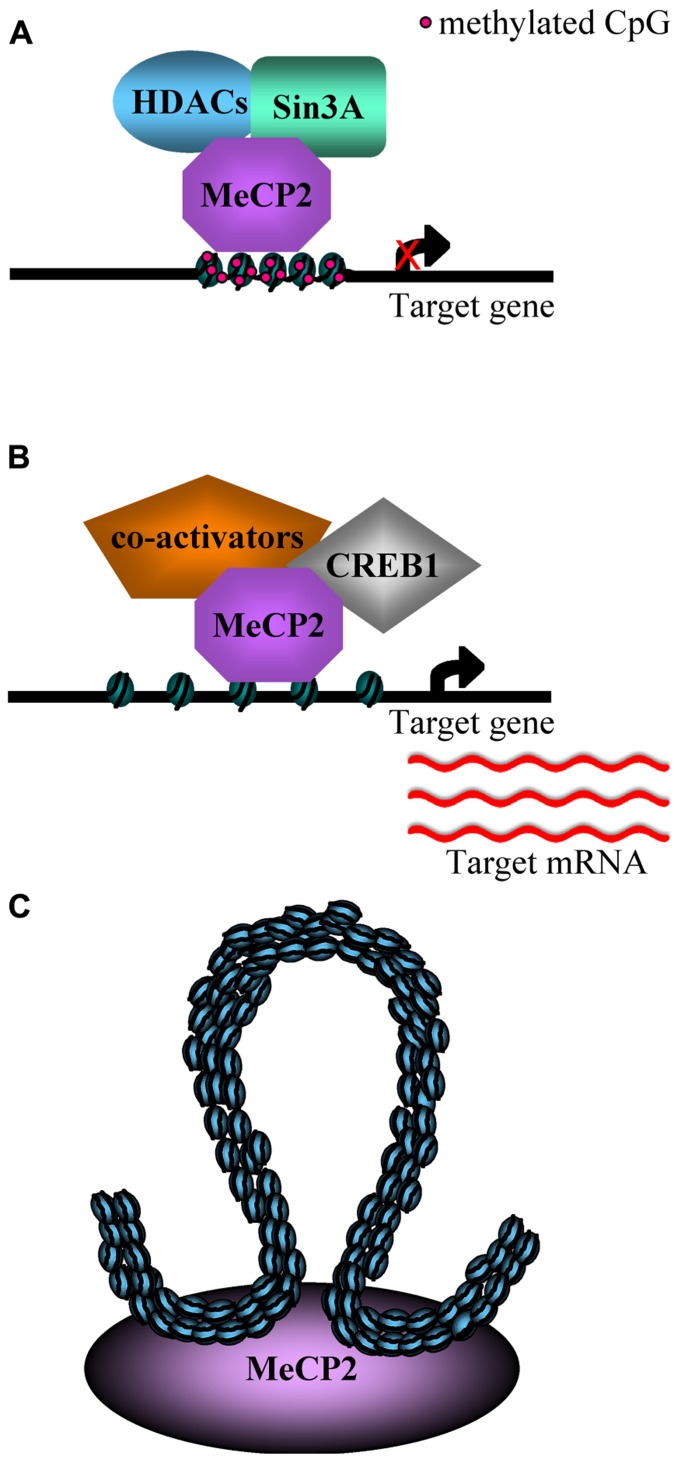
**(A)** An example of MeCP2 mediated transcriptional repression: the methyl-DNA binding domain (MBD) binds methylated CpG sites and recruits histone deacetylases and co-repressors (Sin3A), inducing chromatin compaction and gene silencing (Nan et al., 1998; Chen et al., 2003; Klose and Bird, 2003). **(B)** MeCP2 is able to activate the transcription of some genes in hypothalamus, by binding unmethylated promoters and recruiting CREB1 and, potentially, co-activators (Chahrour et al., 2008). **(C)** MeCP2 is responsible for the silencing of Dlx5/6 imprinted locus by inducing the formation of a silent higher order chromatin loop (Horike et al., 2005).

To simplify the expression analysis of a complex tissue such as the brain, Zoghbi and colleagues (Chahrour et al., 2008; Ben-Shachar et al., 2009) performed microarray expression analyses, respectively, in the hypothalamus and cerebellum of Mecp2 null mice and of over-expressing mice (MECP2-Tg; Collins et al., 2004), comparing the results with wild type (WT) mice. Surprisingly, both reports revealed that MeCp2 is responsible for a subtle repression but also for an activation of many genes, and that some of them were similarly, deregulated in both hypothalamus and cerebellum of the Mecp2 null and MECP2-Tg mice (**Figures 1A,B**). Furthermore, it has been confirmed that MeCP2 directly binds the promoter region of the genes down-regulated in the Mecp2 null mice and up-regulated in the MECP2-Tg mice, while sequential ChIP assays have revealed that the promoter of the activated genes is simultaneously associated with both MeCP2 and the known transcriptional activator CREB1 (**Figure 1B**). These data suggest that MeCP2 regulates the expression of a wide range of genes in different brain sub-regions and point to a role for MeCP2 as a modulator of transcription that can both activate or repress target genes (Chahrour et al., 2008; Ben-Shachar et al., 2009). Moreover, the transcriptional alterations observed in the MECP2-Tg mice have confirmed the deleterious effect of the Mecp2 over-expression reported by different research groups (Collins et al., 2004; Luikenhuis et al., 2004).

A category of genes investigated as a putative target of MeCP2 is that of imprinted genes, whose expression is regulated by differential methylation. For example, several studies have focused on Ube3A, a gene imprinted in the brain (Rougeulle et al., 1998) and associated with Prader–Willi and Angelman syndromes. However, to date, the expression alteration of this gene in Mecp2-null mice has not been clearly understood (Guy et al., 2011). Another imprinted region bound by MeCP2 in the mouse brain includes the Dlx5 and Dlx6 genes, located in an imprinted gene cluster on chromosome 6. Its transcription is nearly two times greater in brains of Mecp2-null mice compared to those of WT mice and, in the same model, the chromatin loop in the Dlx5/6 locus enriched with methylated H3K9 present in the WT brain is absent (**Figure 1C**; Horike et al., 2005).

MeCP2 deficiency affects also Line-1 (L1) transcription and retrotransposition: these are, in fact, increased in the mouse brains from null mice and in the neural precursor cells obtained from iPSC and postmortem brains from RTT patients (Muotri et al., 2010).

The L1 elements are retrotransposons representing 20% of mammalian genomes that may induce genomic alterations, such as insertions and deletions (Kazazian, 1998; Perepelitsa-Belancio and Deininger, 2003; Han and Boeke, 2004). Moreover, a massive somatic L1 insertion can occur in adult brain tissues, a phenomenon that can alter the expression of the neuronal genes (Muotri et al., 2005; Coufal et al., 2009). These data were confirmed by another report which revealed an increased transcript level of the L1-elements, intracisternal A particles, and tandem repetitive units of the mouse major satellite in the Mecp2 null brains compared to WT mice (Skene et al., 2010).

### MeCP2 AND ITS COFACTORS

As already described regarding the interaction between MeCP2 and CREB, proteins with which it interacts may modify the roles of MeCP2 (**Figures 1A,B**).

The first potential connection between MeCP2 and chromatin came from the finding that MeCP2 copurifies with the Sin3-histone deacetylase complex (Jones et al., 1998; Nan et al., 1998). Based on this observation, most current models depict MeCP2 as a transcriptional repressor that facilitates repression through local histone deacetylation mediated by the passive recruitment of histone deacetylases (Bird and Wolffe, 1999). Klose and Bird (2004) demonstrated that MeCP2 is a non-obligatory component of the Sin3a co-repressor complex. Moreover, MeCP2 exists as a monomeric protein in solution and does not stably associate with other proteins.

In addition to Sin3a, several other factors have been reported to bind mammalian MeCP2, including DNMT1, CoREST, Suv39H1, and c-SKI (Nan et al., 1998; Kokura et al., 2001; Lunyak et al., 2002; Kimura and Shiota, 2003) although the contribution of these factors to MeCP2-mediated repression is not known.

MeCP2 also interacts with ATRX, a SWI/SNF family ATPase. MeCP2 recruits ATRX to the heterochromatic foci, but this localization is disrupted in Mecp2 null neurons. ATRX localization is disrupted also by the A140V MECP2 mutation found in XLMR patients (Orrico et al., 2000; Nan et al., 2007). Unexpectedly, the complex MeCP2/ATRX with cohesin preferentially binds the unmethylated allele of the H19 gene. This may depend on the association of MeCP2 with this large complex or on regions of non-specific affinity present in MeCP2 (Guy et al., 2011).

A binding of MeCP2 to the trithorax-related protein Brahma (Brm) has also been reported. Brm and MeCP2 assemble on the methylated genes involved in cancer and on the FMR1 gene in fragile X syndrome (Harikrishnan et al., 2005). Therefore, this interaction is still controversial (Hu et al., 2006).

A physical interaction between the heterochromatin protein 1 (HP1) and MeCP2 has been demonstrated during the myogenic differentiation. In particular, this interaction leads to the re-localization of HP1γ to the heterochromatin, which correlates with the presence of MeCP2 (Agarwal et al., 2007). There is no doubt that works aimed at the dissection of the interactions of MeCP2 with other partners, in particular using the novel sequencing-based techniques (Skene et al., 2010), may open the way to a better understanding of the roles and functions of MeCP2.

### MeCP2: GLOBAL REGULATORY ROLES

**FIGURE 2:**
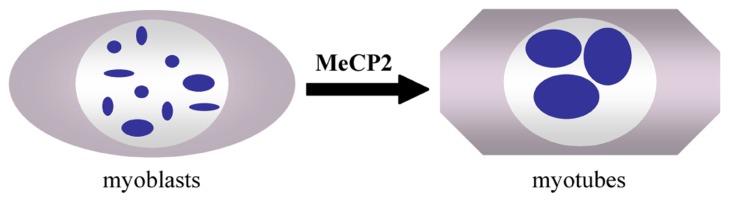
**Clustering of pericentromeric heterochromatin domains (chromocenters, blue spots) during myogenic differentiation of C2C12 myoblasts to myotubes (Brero et al., 2005)**.

DNA methylation affects the nuclear architecture, as measured by the gene position alterations in the chromosome territories (Matarazzo et al., 2007). A direct role of MeCP2 in nuclear architecture rearrangements has not been reported. Rather, the role(s) of MeCP2 in genome-wide phenomena, such as pericentromeric heterochromatin clustering, has recently been analyzed (Brero et al., 2005; Agarwal et al., 2011; Singleton et al., 2011). During the myogenic differentiation of mouse C2C12 cells, the pericentric heterochromatin domains undergo a reorganization and cluster into a smaller number of larger chromocenters (**Figure 2**). These events are accompanied by an increase in the methylation of major satellite DNA and the accumulation of MeCP2 and MBD2 proteins in the nuclei of terminally differentiated muscle cells. Interestingly, the over-expression of MeCP2 and MBD2 in C2C12 myoblasts in the absence of differentiation also induces an aggregation of the chromocenters, indicating that these proteins may be directly involved in the reorganization of heterochromatin architecture. Moreover, studies in Mecp2 null mouse neurons have revealed significant differences in the number and size of the nucleoli and chromocenters compared to WT animals (Singleton et al., 2011). Already in 2002, it was shown that mice carrying a Mecp2 truncating mutation have a higher level of hyperacetylated histone H3 compared with WT mice, emphasizing a generally altered chromatin architecture (Shahbazian et al., 2002). The development of techniques permitting genome-wide epigenomic studies are contributing to the assessment of MeCP2 functions in the chromatin architecture and genome organization.

In 2007, LaSalle and colleagues reported, by ChIP-chip analysis on a neuroblastoma cell line, that more than half of the MeCP2 binding sites are intergenic and that only a small number of them reside in the CpG islands. Moreover, among binding sites located in the CpG islands, many of them are associated with actively transcribed genes, supporting the view of a more complex function of MeCP2 (Yasui et al., 2007).

Different approaches, reagents, and technologies led, some years later, to the re-establishment of MECP2 as a protein with a global regulatory role (Skene et al., 2010). The utilization of next generation sequencing approaches in the neuronal nuclei from the mature mouse brain has revealed that the abundance of MeCP2 is similar to the number of nucleosomes (Skene et al., 2010). Moreover, as previously reported (Shahbazian et al., 2002), in the absence of Mecp2, the H3 acetylation levels are increased, while the H1 levels are doubled, pointing a role for MeCP2 in the global chromatin organization.

Furthermore, an analysis of binding sites around known regulated genes, such as BDNF and Dlx5/6, transcriptionally active in this cellular system, has revealed a MeCP2 binding across the entire locus, except for the CpG island regions, suggesting that these active promoters are unable to bind MeCP2 due to its hypomethylation state. Moreover, high-throughput data suggest that the MeCP2 binding *in vivo* tracks the density of methyl-CpG in the genome (Skene et al., 2010). These latter data have revealed that MeCP2 is one of the most abundant nuclear proteins in the mature neurons suggesting a crucial role for MeCP2 in neurons as a regulator of the entire genome.

The described data suggest that, in addition to the role of MeCP2 as a gene-specific transcriptional regulator, mediated by the association with specific cofactors, the global chromatin-binding function of MeCP2 is crucial for global chromatin dynamics especially during brain maturation. MeCP2 may thus be seen as a multifunctional and structural organizing factor. Furthermore, the interaction of MeCP2 with most regions of the genome, such as the intergenic DNA and repetitive elements, should contribute to keep the rate of somatic mutation and transcriptional noise in the brain low and allows to hypothesize further pathogenic roles for MeCP2 in RTT. This evidence recalls the concept we previously described, focusing on the role of MeCP2 as a key player in genome architecture and regulation.

## Conflict of Interest Statement

The authors declare that the research was conducted in the absence of any commercial or financial relationships that could be construed as a potential conflict of interest.
